# Influence of Composite Type, Surface Treatment, and Thermocycling Aging on the Shear Bond Strength of Repaired Composite Resins

**DOI:** 10.3290/j.jad.c_2659

**Published:** 2026-04-30

**Authors:** Didar Dilan Hartavi, Rafat Sasany, Murat Tiryaki

**Affiliations:** a Didar Dilan Hartavi Assistant Professor, Department of Restorative Dentistry, Biruni University, Faculty of Dentistry, İstanbul, Turkey. Conceptualization, experimental design, manuscript drafting, and proofreading.; b Rafat Sasany Associate Professor, Department of Prosthodontics, Biruni University, Faculty of Dentistry, Istanbul, Turkey. Investigation, manuscript revision and editing, and correspondence.; c Murat Tiryaki Assistant Professor, Department of Restorative Dentistry, Istanbul University, Faculty of Dentistry, İstanbul, Turkey. Supervision, data interpretation, discussion development, and manuscript review.

**Keywords:** dental adhesion, composite resin repair, shear bond strength, surface treatment, thermocycling

## Abstract

**Purpose:**

This study evaluated the effects of different surface treatment methods and thermocycling on the shear bond strength (SBS) of repaired composite resin restorations across three composite types (nanohybrid, bulk-fill, and microhybrid).

**Methods and Materials:**

A total of 252 disk-shaped specimens (Ø20 × 5 mm, n = 7 per group) were fabricated from three composite resins: a nanohybrid composite (Filtek Z550; 3M ESPE), a bulk-fill composite (Opus Bulk Fill; FGM), and a microhybrid composite (Gradia Direct Anterior; GC). The specimens were divided into four subgroups according to surface treatment: no surface treatment (ST-C), airborne-particle abrasion (ST-AO), bur roughening with a medium-grit diamond bur under water cooling (ST-B), and phosphoric acid etching followed by application of a universal adhesive (ST-PH). For all groups, the repair procedure was performed using a nanohybrid composite (Filtek Z550; Ø2.38 × 3 mm). Aging protocols included water storage at 37°C for 24 h (Immediate), thermocycling for 10,000 cycles, and 30,000 cycles between 5°C and 55°C. Shear bond strength (SBS) was measured using a universal testing machine at a crosshead speed of 1 mm/min. Data were analyzed using the Shapiro–Wilk and Levene tests, followed by three-way ANOVA (with stratified one-way ANOVAs where interactions were significant) and Tukey post hoc tests (α = 0.05).

**Results:**

A three-way ANOVA revealed that composite type, surface treatment, and aging condition significantly affected SBS (*P* < 0.001). SBS differed significantly among composites and surface treatments (*P* < 0.001). For RC-BF, ST-PH provided the highest bond strength after 10,000 and 30,000 cycles. In RC-MH, bur roughening (ST-B) yielded the highest bond strength at 10,000 cycles. For RC-NH, ST-PH initially showed the highest SBS values; however, at 30,000 cycles, ST-B provided the best repair stability for the RC-NH group (12.96 MPa). However, the highest overall SBS value across all tested materials at this final aging stage was achieved by the RC-BF group treated with ST-PH (13.91 MPa).. Thermocycling significantly reduced SBS (*P* < 0.001). Nanohybrid composites exhibited relatively stable SBS values across aging conditions.

**Conclusion:**

Appropriate surface treatment, particularly phosphoric acid etching combined with universal adhesive application, significantly increased shear bond strength following thermocycling. Nanohybrid composites demonstrated stable SBS trends across aging conditions compared with the other materials.

The advancement of adhesive dentistry has reinforced the importance of minimally invasive treatment approaches, favoring the repair of defective restorations over complete replacement.^1^ This philosophy preserves the natural tooth structure, enhances restoration longevity, and improves overall oral function. A critical determinant of successful composite resin repair is the bond strength between the existing restoration and the repair material. Inadequate adhesion can lead to debonding, marginal failure, or reduced mechanical performance, compromising clinical longevity. Consequently, optimal surface treatment and material selection are essential to achieving a strong and durable repair interface.^13,20,21,23^

Composite resins are widely used for direct and indirect restorations due to their excellent esthetic properties, mechanical strength, and minimally invasive application. However, despite advancements in material formulations, restorations remain prone to fractures, chipping, and marginal degradation, necessitating repair rather than replacement.^9^ The key challenge in composite repair is the absence of an oxygen-inhibited layer on aged composite surfaces, which limits chemical bonding.^22,32^

To overcome this limitation, various surface treatments, both mechanical (bur roughening, sandblasting) and chemical (bonding agents, silane application), have been investigated to enhance repair bond strength.^2,15,16,17,28^ Studies have shown that surface roughness significantly affects adhesion, with combined mechanical and chemical approaches generally yielding superior bond strength compared to bonding agents alone.^11,31^ These methods remove the superficial composite layer, introduce surface irregularities, and improve the wettability of bonding agents. Additionally, silane coupling agents and low-viscosity adhesives facilitate chemical bonding by enhancing resin penetration into the roughened surface.^6,14,18,24,26,27,30^

The composition of the original and repair materials also influences the performance of composite repairs. In this study, three types of composites were examined: nanohybrid, bulk-fill, and microhybrid.^3,4,7^ Nanohybrid composites offer a balance of mechanical strength and esthetics, bulk-fill composites are designed for efficient application and deeper curing, and microhybrid composites provide superior polishability and wear resistance.^4,7^ Understanding how these different composites interact in repair situations is critical for improving clinical outcomes.

Another factor affecting repair bond strength is intraoral aging, which involves water absorption, resin component leaching, and enzymatic degradation.^24^ In laboratory studies, thermocycling is commonly used to replicate the thermal stresses encountered in the oral environment and assess the longevity of bonded interfaces.^8,12^ In this study, shear bond strength was tested immediately after bonding and after 10,000 and 30,000 thermocycles to simulate different levels of aging.

Therefore, this study aimed to evaluate the effects of composite type, surface treatment method, and thermocycling aging on the shear bond strength of composite resin repairs. The null hypotheses tested were: (1) composite type does not significantly affect shear bond strength; (2) surface treatment method does not significantly affect shear bond strength; and (3) thermocycling aging does not significantly affect shear bond strength.

## METHODS AND MATERIALS

**Fig 1:**
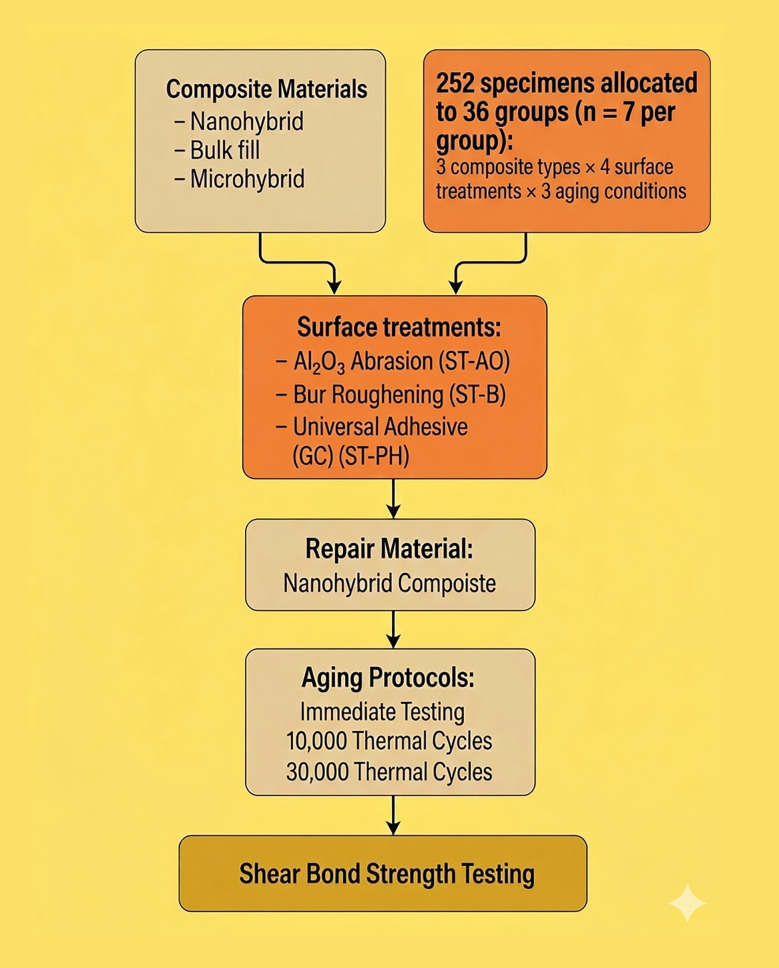
Flowchart illustrating the experimental design.

A total of 252 specimens were prepared and allocated according to a full-factorial design consisting of three composite types, 4 surface treatment protocols, and three aging conditions (n = 7 per subgroup). Each composite group (n = 84) was subdivided into four surface treatment groups (n = 21), and each surface treatment group was further divided into three aging subgroups (Immediate, 10,000 cycles, and 30,000 cycles; n = 7 per aging condition). The experimental design is illustrated in Figure 1.

A wooden mold (Ø14 × 20 mm) was fabricated and embedded in polyvinyl siloxane impression material (Affinis; Coltene Whaledent, Altstätten, Switzerland) to create silicone molds. Using these molds, 252 acrylic blocks (Ø14 × 20 mm) were prepared as standardized specimen bases. Composite disks were fabricated separately and positioned centrally on the acrylic blocks during specimen preparation. No intermediate adhesive or bonding agent was used between the composite substrate and the acrylic base in order to eliminate potential confounding bonding variables. An overview of the materials used in this study is provided in Table 1. Three composite substrates were used (n = 84 each):

**Table 1:** Composite materials used in the study

Composite code	Composite type	Brand name	Organic matrix	Inorganic phase
RC-BF	Bulk-fill	Opus Bulk Fill Composite (FGM; Joinville, Brazil)	UDMA, stabilizers, photoinitiators	Silicon dioxide, pigments
RC-MH	Microhybrid	Gradia Direct Anterior Composite (GC Corp.; Tokyo, Japan)	UDMA, camphorquinone	Microfine prepolymerized silica fillers
RC-NH	Nanohybrid	Filtek Z550 Universal Nanohybrid Composite (3M ESPE; St. Paul, MN, USA)	UDMA, Bis-GMA	Silica/zirconia nanofillers

Group RC-NH: Filtek Z550 Universal Nanohybrid Composite (3M ESPE; St. Paul, MN, USA)
Group RC-BF: Opus Bulk Fill Composite (FGM; Joinville, Brazil)
Group RC-MH: Gradia Direct Anterior Microhybrid Composite (GC; Tokyo, Japan)

Specimens were assigned to one of three experimental surface treatments:

1. Bur roughening (ST-B): A medium-grit diamond bur (Meisinger 805; Neuss, Germany) was applied under continuous water cooling for 10 s using a standardized handpiece protocol. All procedures were performed by a single experienced operator, and burs were replaced after every five uses.
2. Airborne-particle abrasion (ST-AO): Airborne-particle abrading with 50 µm Al_2_O_3_ at 120 psi for 10 s from a 10 mm distance, followed by ultrasonic cleaning in distilled water for 5 min.
3. Phosphoric acid + adhesive (ST-PH ): Etching with 37% phosphoric acid gel (FGM; Joinville, Brazil) for 15 s, rinsing for 15 s, and air-drying for 5 s. A universal adhesive (G-Premio Bond; GC; Tokyo, Japan) was applied for 10 s, gently air-dried for 5 s, and light-cured for 10 s using an LED unit (1000 mW/cm²; Demetron 2, Kerr; Middleton, WI, USA). This adhesive protocol was performed exclusively in the ST-PH group. No adhesive was applied in the ST-B, ST-AO, or ST-C groups.

All repairs were performed using Filtek Z550 Universal Nanohybrid Composite (3M ESPE; St. Paul, MN, USA). A cylindrical Teflon mold (Ø2.38 × 3 mm) was positioned on the treated surfaces, filled with repair composite, and light-cured for 20 s (1000 mW/cm^2^).

All surface treatment groups, including the untreated control group (ST-C), were divided into three subgroups according to aging condition: specimens were aged by water storage at 37°C for 24 h (Immediate) or subjected to thermocycling (Modental; Esetron Mekatronik, Ankara, Turkey) for 10,000 or 30,000 cycles between 5°C and 55°C with 20 s dwell time and 10 s transfer time to simulate oral aging.

SBS was measured using a universal testing machine (Shimadzu AG-X; Kyoto, Japan). A cylindrical steel rod (Ø2 mm) applied force at 90 degrees to the bonded interface at a crosshead speed of 1 mm/min until fracture. Bond strength was recorded in MPa.

Fracture surfaces were examined under a stereomicroscope (SZ-61; Olympus, Tokyo, Japan) at ×40 magnification and classified as adhesive, cohesive, or mixed. Selected specimens were further examined using a scanning electron microscope (JCM-5000 NeoScope; JEOL, Tokyo, Japan) at magnifications of ×100 and ×200.

### Statistical Analysis

*A priori* power analysis was conducted using G*Power software (Version 3.1; Heinrich-Heine-Universität Düsseldorf, Germany) for a three-way ANOVA design (3 composite types × 4 surface treatments × 3 aging conditions). Assuming an effect size of f = 0.25, a significance level of α = 0.05, and a statistical power of 0.95, the required total sample size was calculated as 252 specimens (n = 7 per subgroup). The analysis was structured to detect both main and interaction effects.

Statistical analyses were performed using IBM SPSS Statistics 22 (IBM, Armonk, NY, USA). Data normality was assessed using the Shapiro–Wilk test and homogeneity of variances using Levene’s test. A three-way ANOVA was conducted to evaluate the effects of composite type, surface treatment, and aging condition, as well as their interactions, on SBS. When significant interactions were identified, simple main effects were analyzed. Post hoc pairwise comparisons were performed using Tukey’s HSD test. Effect sizes were reported as partial η^2^. Statistical significance was set at α = 0.05. Failure mode distributions were analyzed using the Chi-square test.

## RESULTS

**Fig 2:**
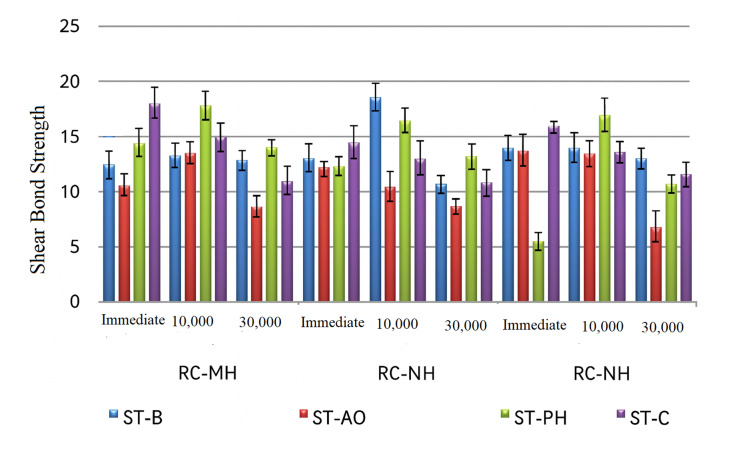
Mean shear bond strength (MPa) of repaired composite resins subjected to various surface treatments and different aging protocols.

Significant differences in shear bond strength were observed among the composite materials (three-way ANOVA, *P* < 0.001, partial η^2^ = 0.42; Table 2). In the immediate groups, RC-NH and RC-MH showed significantly higher bond strength than RC-BF (Tukey HSD, *P* < 0.05; Table 3). After 10,000 thermocycles, RC-MH treated with bur roughening (ST-B) exhibited the highest bond strength (18.57), while RC-BF with ST-PH also showed comparably high values (17.74). At 30,000 cycles, RC-BF with ST-PH demonstrated the highest overall bond strength (13.91 MPa). Within the RC-NH group specifically, ST-B yielded the highest values (12.96 MPa) at this advanced aging stage, showing better resistance to degradation than other RC-NH protocols; (*P* < 0.05, Table 3, Fig 2).

**Table 2:** Three-way ANOVA results evaluating the effect of composite type, surface treatment, and aging on shear bond strength

Source	Type III Sum of Squares	df	Mean Square	F	*P* value
Composite	110.267	2	55.133	45.454	*P* < 0.001
Surface preparation	411.893	3	137.298	113.193	*P* < 0.001
Aging	590.078	2	295.039	243.24	*P* < 0.001
Composite × Surface preparation	163.331	6	27.222	22.443	*P* < 0.001
Composite × Aging	60.153	4	15.038	12.398	*P* < 0.001
Surface preparation × Aging	482.015	6	80.336	66.232	*P* < 0.001
Composite × Surface preparation × Aging	428.706	12	35.726	29.453	*P* < 0.001


**Table 3:** Shear bond strength values (MPa) according to surface treatment in different composite and aging groups

Composite resin	Aging	ST-B	ST-AO	ST-PH	ST-C
RC-BF	Immediate group	18.06 ± 1.20^a^	10.54 ± 0.99^b^	14.41 ± 1.26^c^	18.04 ± 1.35^a^
	10,000	13.91 ± 0.89^a^	13.47 ± 0.76^a^	17.74 ± 1.32^b^	14.90 ± 1.29^c^
	30,000	12.98 ± 0.86^a^	8.58 ± 1.04^b^	13.91 ± 0.70^b^	10.92 ± 1.25^c^
RC-MH	Immediate group	12.96 ± 1.23^a^	12.09 ± 0.95^a^	13.22 ± 0.74^b^	14.31 ± 1.47^c^
	10,000	18.57 ± 1.36^a^	13.91 ± 1.11^b^	16.31 ± 1.38^c^	12.71 ± 1.69^a^
	30,000	10.76 ± 0.72^a^	9.50 ± 1.16^b^	10.61 ± 1.25^a^	8.76 ± 1.47^b^
RC-NH	Immediate group	13.94 ± 1.25^a^	13.64 ± 1.22^a^	16.91 ± 1.48^b^	15.57 ± 0.74^c^
	10,000	13.91 ± 1.11^a^	5.48 ± 0.67^b^	16.91 ± 1.48^c^	10.90 ± 1.35^d^
	30,000	12.96 ± 0.89^a^	6.72 ± 1.49^b^	10.62 ± 0.55^c^	11.36 ± 1.05^c^
Values are presented as mean ± standard deviation (MPa). Different superscript letters within the same row indicate significant differences among surface treatments according to Tukey’s HSD post hoc test (*P* < 0.05). RC-BF = bulk-fill composite resin; RC-MH = microhybrid composite resin; RC-NH = nanohybrid composite resin; ST-B = bur roughening; ST-AO = air abrasion with Al_2_ O_3_; ST-PH = phosphoric acid etching; ST-C = control (no treatment).					

Surface treatment significantly affected bond strength (*P* < 0.001, partial η^2^ = 0.37). ST-PH consistently produced the highest values for RC-BF and RC-NH, while ST-B was superior in RC-MH at 10,000 cycles. ST-AO yielded the lowest bond strength across all conditions (*P* < 0.05). Overall, ST-PH and ST-B outperformed ST-AO, particularly after thermocycling.

Thermocycling significantly reduced bond strength overall (*P* < 0.001, partial η^2^ = 0.45). In the immediate groups, ST-PH and ST-C exhibited the highest values. After 10,000 cycles, RC-MH treated with ST-B and RC-BF treated with ST-PH exhibited the highest SBS values within their respective groups. At 30,000 cycles, SBS values further decreased, with ST-AO showing the greatest reduction. Within the RC-NH group, ST-B demonstrated the highest SBS at this aging stage, whereas the overall highest single value was observed in RC-BF treated with ST-PH (13.91) (Table 3, Fig 2).

**Fig 3a to i:**
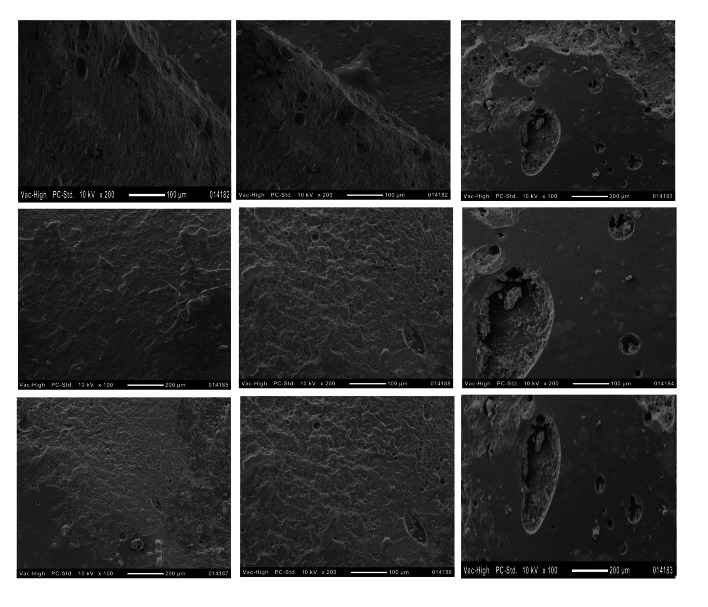
Representative SEM images of failure modes observed in different composite groups. The top row shows RC-BF (Bulk-Fill composite), the middle row shows RC-MH (Microhybrid composite), and the bottom row shows RC-NH (Nanohybrid composite). Each row presents three distinct failure types: (a, d, g) Cohesive failure, where the fracture occurs within the composite material; (b, e, h) Adhesive failure, where separation occurs at the bonding interface; and (c, f, i) Mixed failure, showing characteristics of both adhesive and cohesive failure patterns.

A total of 72 specimens were evaluated for failure mode analysis following shear bond testing. Failure types were classified as adhesive, cohesive, or mixed under stereomicroscopic examination (×40). RC-BF predominantly exhibited cohesive failures, whereas RC-MH demonstrated mainly adhesive failures. RC-NH showed a more balanced distribution of cohesive and mixed failures. The distribution of failure modes differed significantly among composite types (Chi-square test, *P* < 0.05) (Table 4). Representative SEM images confirmed the observed fracture patterns (Fig 3).

**Table 4:** Distribution of failure modes Distribution of failure modes among different composite types

Failure mode	RC-BF (n = 24)	%	RC-MH (n = 24)	%	RC-NH (n = 24)	%
Adhesive	2	8.3%	16	66.7%	4	16.7%
Cohesive	16	66.7%	2	8.3%	12	50.0%
Mixed	6	25.0%	6	25.0%	8	33.3%
Total	24	100%	24	100%	24	100%
Values are presented as a number (percentage). Failure modes were classified as adhesive, cohesive, or mixed. Differences in failure mode distributions among groups were analyzed using the Chi-square test (*P* < 0.05). RC-BF = bulk-fill composite resin; RC-MH = microhybrid composite resin; RC-NH = nanohybrid composite resin.						

## DISCUSSION

This study evaluated the influence of composite type, surface treatment method, and thermocycling aging on the repair bond strength of composite resins. The findings demonstrated that all three factors significantly affected bond performance, leading to the rejection of the null hypotheses.

The etch-and-rinse mode of the universal adhesive was applied only in the ST-PH group. Although this approach is not universally superior in all clinical situations, it has been recommended for certain universal adhesives and was adopted here to evaluate its effect in combination with phosphoric acid etching.^6,14,28^ Surface roughening, particularly with a diamond bur, has repeatedly been emphasized as a critical factor for improving micromechanical retention by increasing surface irregularities and enhancing adhesive penetration.^26,28^ Consistent with previous findings, the present results demonstrated that ST-B and ST-PH yielded the highest repair bond strengths, while ST-AO consistently exhibited the lowest values.

The aging of composite resins in the oral environment is associated with hydrolytic degradation, leaching of resin components, and debonding at the filler–matrix interface.^31^ Thermocycling is widely used to simulate these processes, with 10,000 cycles approximating one year of intraoral service.^6,28^ In this study, bond strength decreased progressively after 10,000 and 30,000 cycles, confirming that thermal fatigue significantly compromises adhesive interfaces. These results are consistent with previous reports that bond durability declines with aging, even when optimal surface treatments are applied.^13–26^

The performance of different composite substrates also influenced repair outcomes. RC-NH and RC-MH exhibited superior bond strength in the immediate groups, whereas RC-BF showed improved performance after 10,000 cycles. At 30,000 cycles, RC-NH demonstrated the best stability, while RC-MH exhibited the steepest decline. This can be explained by differences in material composition: the dense filler network and low-shrinkage resin matrix of nanohybrid composites may contribute to their stability, while the UDMA-based resin matrix in microhybrid composites may be more susceptible to hydrolytic degradation.^7,20^ The comparatively better performance of bulk-fill composites after aging could be attributed to their modified resin matrix designed for deeper curing and stress relief.^4^

Regarding failure modes, most specimens demonstrated cohesive failure within the repair composite, particularly in RC-NH after thermocycling. This indicates that adhesion at the interface exceeded the internal strength of the material. In contrast, the ST-AO group showed a high frequency of adhesive failures, highlighting its limited bonding capability. This provides strong evidence that the parameters used for air abrasion in this study (particle size, pressure, nozzle distance) may not have been optimal, which likely explains the discrepancy with previous studies reporting superior outcomes for this technique.

Comparison with previous studies further supports these findings. Ozcan et al reported comparable repair bond strength for microhybrid and nanohybrid composites after bur roughening, although microhybrid composites generally displayed more favorable outcomes due to reduced polymerization shrinkage from broader particle size distribution.^23^ Other studies have shown that air abrasion followed by adhesive application can yield high bond values,^2,13^ but in this study, ST-AO performed poorly, likely due to the formation of a smear layer and limited adhesive penetration into surface irregularities.

The effect of using the same versus different repair composites remains controversial. Some authors recommend identical materials to ensure chemical compatibility,^26^ while others emphasize that clinical identification of composite type is often impractical.^24^ In this study, the same nanohybrid composite was used as the repair material across all groups to minimize confounding factors. However, results demonstrated that identical resin chemistries did not necessarily improve bond strength, in line with recent studies suggesting that substrate properties and surface treatment protocols play a more decisive role.^22,25,29^

The outcomes of this study highlight that successful composite repair cannot be attributed to a single factor in isolation. Instead, the interplay between the composite type, the surface treatment method, and the degree of aging emerged as a critical determinant of long-term bond durability. This suggests that a comprehensive understanding of material behavior under aging conditions is essential to optimize clinical protocols. In particular, the superior performance of nanohybrid composites subjected to combined mechanical and chemical surface conditioning even after prolonged thermocycling emphasizes the importance of a tailored, multifactorial approach rather than a standardized repair protocol. These findings underscore the need for clinicians to consider not only the restorative material in use but also the patient-specific intraoral conditions when selecting repair strategies.

This study was performed under *in vitro* conditions, which do not fully replicate the complex biological and mechanical environment of the oral cavity. Only three composite substrates and a single nanohybrid repair composite were evaluated, limiting the generalizability of the findings to other restorative materials. Thermocycling was used as the sole aging method, without incorporating cyclic mechanical loading from mastication or salivary contamination, which are additional critical variables in the oral environment and can significantly affect bond durability. Additionally, only one adhesive protocol was investigated, and alternative bonding strategies or repair materials were not considered. These factors should be addressed in future studies to provide a more comprehensive understanding of the clinical performance of composite repairs.

## CONCLUSION

Composite type, surface treatment protocol, and aging condition significantly influenced repair shear bond strength. The effects of phosphoric acid etching combined with a universal adhesive (ST-PH) and bur roughening alone (ST-B) varied depending on the composite material and aging level. Airborne-particle abrasion (ST-AO) generally resulted in lower bond strength values. Thermocycling significantly reduced shear bond strength across groups. Among the tested composites, nanohybrid composites (RC-NH) exhibited relatively stable performance over aging, whereas microhybrid composites (RC-MH) showed the greatest reduction over time. Failure mode analysis revealed that cohesive failures within the repair composite were predominant, indicating that the adhesive interface often resisted stress better than the material itself.

### Clinical Relevance

The SBS values of the most successful protocols in this study, particularly ST-PH treatment in the nanohybrid composite, exceeded commonly accepted clinical threshold values (6–8 MPa). This confirms that the evaluated protocols are not only statistically but also clinically effective, reinforcing their practical applicability in restorative dentistry.
